# Research ethics oversight in Norway: structure, function, and challenges

**DOI:** 10.1186/s12913-018-3816-0

**Published:** 2019-01-10

**Authors:** R. Froud, T. J. Meza, K. O. Ernes, A. M. Slowther

**Affiliations:** 1 Department of Health Sciences, Kristiania University College, Oslo, Norway; 2 Department of Management and Organization, Kristiania University College, Oslo, Norway; 3 Warwick Medical School, University of Warwick, Coventry, UK

**Keywords:** Ethics, Health services research, Clinical trials, Undergraduate research

## Abstract

**Background:**

While the development and evaluation of clinical ethics services in Norway has been recognized internationally, the country’s research ethics infrastructure at times may have been less well developed. In 2016, media interest in the controversial nature of some health services research and pilot studies highlighted gaps in the system with certain types of research having no clear mechanisms through which they may be given due independent consideration. It is not clear that new legislation, implemented in 2017, will address this problem.

**Summary:**

We explore relevant law, committee scope, and the function of the system. We show that 1) Norwegian law provides for ethics assessment for all forms of health research; 2) regional RECs in Norway might not have always enforced this provision, considering some interventional health services research to be outside their remit; and 3) Norwegian law does not explicity provide for local/university RECs, meaning that, in practice, there may be no readily accessible mechanisms for the assessment of research that is excluded by regional RECs. This may include health services research, pilot studies, and undergraduate research. New 2017 legislation has no effect on this specifically but focuses on institutions regulating researcher activity. This may place researchers in the difficult situation of on one hand, needing to hold to recognized ethical standards, while on the other, not readily having access to independent committee scrutiny to facilitate consistent operation with these standards.

**Conclusion:**

To support researchers in Norway and to protect the public, it may be necessary either to widen the regional RECs’ remit or to make legislative alterations that permit and do not discourage the existence of local RECs.

## Background

In 1963, three doctors at the Jewish Chronic Disease Hospital, in Brooklyn, New York, injected cancer cells into 22 patients without their knowledge or consent [1]. The study was conducted without institutional review, but with the funding of the US Public Health Service, and the American Cancer Society [2]. Following litigation by several doctors, the New York Board of Regents decided that the study violated medical ethics [1]. In 1964, during a period of heightened debate, the UK Royal College of Physicians published a statement recommending that all human research be subject to ethics review, and the World Medical Association adopted the Declaration of Helsinki (the Declaration) [3, 4].

In 1975, the first revision of the Declaration advocated the use of independent committees, which proceeded to proliferate across the US and Europe [5]. In Norway, a Norwegian Council of Medical Research working group proposed a system comprising four RECs, each responsible to an overseeing appeal body and devolving assessments to local RECs (LRECs).^1^ However, the Ministry of Social Affairs considered the proposal too intricate [6]. In 1978, the Research Council of Norway and the Council of Medical Research established a committee with a mandate to cover all research pertaining to human health, as required by the Declaration. In addition, the largest hospitals established their own LRECs.

Since the 1980s, the field of bioethics, and medical ethics in particular, has flourished in Norway, producing some of the leading figures in international bioethics research in areas such as resource allocation and clinical ethics support [7, 8]. Clinical ethics committees are mandated in Norwegian hospitals and there is an extensive program of work supporting ethics deliberation in community health care settings. However, the main focus for development of a research ethics infrastructure has been through legislation and regulation.

In the early 1980s, a lack of clarity regarding funding and organization, threatened the Norwegian research ethics system, which was criticized as lagging behind other Nordic countries [9]. In 1985, the Ministry of Culture and Education established Medical and Health Research RECs and, in 2006, following adoption of the Research Ethics Act, these were provided with ring-fenced funding [10]. The aim of the 2006 Research Ethics Act (which came into force in 2007) was to ensure that research carried out by public and private institutions is conducted in accordance with recognized ethical standards. This legislation, which would later be repealed by the 2017 Act (below), provided for national and regional ethics committees and established their jurisdiction over research projects being conducted in Norway on human subjects, and provided a mechanism for appeals to the national committee in the case of rejected applications, and a commission for the investigation of scientific misconduct.

In 2008, the Health Research Act was passed and came into force in 2009 [11]. The aim of the 2008 Act is to *promote good ethically sound medical and health research* (Clause §1) and applies to *all medical and health research on human beings, human biological material or personal health data, including pilot studies and experimental treatments* (§2) [11]. It has been the role of Norway’s regional RECs to enforce this Act. The 2008 Act outlines a legal requirement for a protocol describing research within its scope (§6). Clause §9 and §10 provides that an application for prior approval must be submitted together with the protocol to the regional committee for medical and health research ethics. In §4, ‘medical and health research’ is defined as ‘*activity conducted using scientific methods to generate new knowledge about health and disease’*. Thus, since the 2008 Act came into force in 2009, the law has been explicit that all Norwegian medical and health research (*i.e*. research that generates new knowledge about health and disease) on human beings, using human biological material, or personal health data, must have prior approval from one of the seven regional medical and health RECs in Norway.

The system, as it was instituted in 2009, continued unchanged and until new legislation in 2017, which followed several research ethics related scandals that cast the light of the media interest and public attention throughout 2016 onto specific research studies. During these debates certain weaknesses of the system were alluded to by stakeholders, which included the person who led the committee involved in the drafting of the 2006 Act.

The 2017 Research Ethics Act repealed the 2006 Act [12]. The Minstry of Education, Science, and Culture explained that its motivation in proposing the new law was to strengthen the ethical component in Norwegian research through promulgation of the responsibility of individual researchers and research institutions [13]. The new legislation expands on material in the 2006 law, adding researcher and institutional responsibilities and conferring on Norwegian research institutions a duty to follow ethical norms; provide researcher training; assemble committees for managing misconduct, fraudulent cases, and breaches of ethics; and to report any breaches to a national examination committee, for which the new law also provides.

Currently, some confusion may remain surrounding procedures for the assessment of health and medical research in Norway that is considered by the regional committees to be out of their remit. It may not be clear what mechanisms are permitted to be in place for appropriate review of such research [14, 15]. There may be extant gaps in the mechanisms provided by the legal and regulatory infrastructure.

In this paper, we consider relevant law, its interpretation and cases of practice, and possible gaps in the infrastructure and any implications. Our objectives are to: 1) examine the current Norwegian system; 2) stimulate debate on research ethics infrastructure using Norway as an example; and 3) consider desirable directions of travel that may improve the situation in Norway for key stakeholders, including ethics committees, researchers, and the public.

## Analysis

One of the studies that received media scrutiny in 2016, was a study defined as a pilot RCT. The study, which explored Human Papilloma Virus (HPV) screening in female patients, and included 60,000 female participants, was reported as being judged by the regional REC to be exploring implementation rather than generating new knowledge and considered exempt [16, 17]. The REC chair at the time stated her view that the pilot RCT fell outside the wording of the Act:

“[*The principle is that the Health Research Act applies to research on health and disease. So the question is whether this is research that will provide new knowledge about this, or whether it's knowledge of implementing. (…) One can always discuss whether to define a project like this as health services research or quality control. Anyway, both these definitions fall outside the Health Research Act. It is possible to argue that definitions of health services research and quality assurance should be included under one of the points of law, but they are not.]”*’- (Translated from Norwegian by the authors) [17]

However, the person who led the committee that drafted the 2006 Health Research Act, disagreed with the interpretation, explaining that there was no doubt in his view that the pilot RCT constituted research: [17]

“[*I was in no doubt that the project that was presented to the council, fell within the research definition…*. *When I led the Health Research committee, our intention was to embrace more projects like this. We experienced that research was being done in the country disguised as quality assurance or test* (pilot) *treatment. The intention (of the Act) was that all exploration relating to new knowledge should be defined as research – in particular to ensure informed consent, which is an important ethical requirement]”* [17].

The interpretation of the limits of ‘medical and health research on human beings’, and the accompanying caveat in the §4 definition of the 2008 Act (above), that the research must generate ‘new knowledge’, may be central to this debate. We suggest that the purpose of research and scientific inquiry is to explore research objectives and provide answers to research questions. We also suggest that it is axiomatic that the intent of health and medical research is to generate new knowledge, and that the reverse also holds; i.e., that (systematic) exploration with the intention of producing new knowledge, is research.

The HPV RCT does appear very large for a pilot study. Given that it randomized 60,000 patients (*n.b* in 2018 this equates to about 5% of the Norwegian female population between the 34–69 age range of entry into the study); some might consider this more appropriately classified a mega-trial [18, 19]. Around 30 participants per arm is generally thought adequate for estimating standard deviation and meeting typical pilot/feasibility study objectives [20].

In any case, it is clear from §2 of the 2008 Act (above) that its scope does include pilot studies and so notwithstanding debate about the HPV RCT’s classification as pilot, it would not be disqualified from consideration on the basis of it being a pilot study. The distinction made by the REC in its interpretation in this case appears to be that the pilot study was thought to be health services research (HSR), or quality assurance, and these kinds of research are in their judgement not covered by the Act.

The Association for Health Services Research defines HSR as “*the multidisciplinary field of scientific investigation that studies how social factors, financing systems, organizational structures and processes, health technologies, and personal behaviors affect access to health care, the quality and cost of health care, and ultimately our health and well-being. Its research domains are individuals, families, organizations, institutions, communities, and populations”* [21]. HSR encompasses epidemiological and qualitative research approaches, as well as phase III/IV clinical trials and pilot/feasibility studies.

A similar Norwegian HSR study that was a pilot RCT of a national screening program for bowel cancer, was published in the British Journal of Cancer in 2016 [22]. This study received REC consideration and approval [23]. Thus, there may have been variable interpretation of the letter of Norwegian law, affecting what has been done in practice.

Some researchers who seek regional REC approval, and who have been informed that it is outside the remit of the 2008 Act, have queried the situation. For example, Mæland *et al* discuss their experience when applying for ethics approval for their research on work and health, but being informed that such projects are not covered by the Act [15]. In the work and health field, return to work is seen as a health outcome and in this sense trials seeking to improve RTW rates are trials seeking to improve health; *i.e.* and are thus to seeking to generate new knowledge [24]. There are grounds for concern if there is no mechanism for independent assessment of protocols for studies such as these.

There was also media interest in 2016 in research studies following allegations of doping in athletes in Norway. One report alleged researchers applied to the REC for approval for a study that proposed to provide athletes with asthma medications (whether or not athletes had the condition) [25]. However, in this case it was reported that the research had been performed before the ethics application had been considered, and that the REC considered the study to be quality assurance. In the publicly available REC correspondence, the committee describes the project as containing ethically questionable aspects, but concluded the project fell outside of their mandate after considering: 1) whether the project’s purpose is to try to improve the quality of a given treatment; 2) whether the project is to try their own practices against established standards; and 3) whether the project involves manipulating the project participants in a way that would not otherwise be done as part of routine clinical practice and quality assurance [26]. It may not be clear how these criteria relate to the wording of the 2008 Act.

A separate study that involved healthy cross-country athletes, swimmers, and students (including minors) was considered outside of REC remit because the study objective was to compare two different methods of measurement [27]. However, in this case it later emerged that participants had also received asthma medication as part of the study and that this part of the protocol had not been disclosed to the REC [14].

Separately to all these cases of HSR that were under the media spotlight in 2016, we suggest that it may also be unclear what mechanisms exist to cover the assessment of undergraduate research. NOKUT, the department responsible for approving degree courses, promotes engaging students with research early at undergraduate level [28]. The 2008 Act must be considered to govern in cases where the research is medical and health research on human beings (some undergraduate research might genuinely be considered to provide new knowledge); includes biological materials (less likely); or manages personal health data (not at all unlikely). As per the legislation wording quoted above, such protocols then legally require regional REC attention. In practice, whether a protocol is reviewed by a regional REC may depend on subjective judgment about whether such work is thought to contribute new health knowledge, if it not judged to be outside of the Act for some other reason. If an undergraduate project is not considered the supervising educational institution is then placed in a difficult position: there may be no alternative legal mechanism for the projects to have independent committee scrutiny. The legislation – *i.e.* that all medical and health on human beings, human biological material, or personal health data, must be sent to one of the government-appointed regional medical and health research RECs – may prevent the establishment of LRECs, which might otherwise review undergraduate protocols. An institution wishing to err on the side of caution might conclude that it should not permit its students to conduct any primary research other than systematic reviews, where data are already in the public domain and ethics approvals are not required.

For comparison, in the UK, undergraduate institutions usually convene their own RECs to consider health research protocols (including experimental protocols) that fall outside the remit of the national Research Ethics Service [29]. There is a suggestion that departments with this duty also function in Norway albeit under designations such as ‘The Department of Research Support’, or ‘Commission for Research Ethics’ [6]. It is not clear whether all research institutions or universities have such departments nor if they are legally permitted to function as ethics committees. If appropriate ethics infrastructure is not in place, experimental student undergraduate research may pose risk to public health. There would be no mechanism for students/supervisors to publish results in any reputable journals, as editors of journals may not view the aforementioned internal departments as qualifying independent ethics committees.

### Implications for health services researchers working in Norway

Most internationally-ranked journals insist upon prior ethics approval for any health research involving the collection of human data in accordance with the Vancouver Convention [30]. If researchers in Norway are unable to gain the prior views of RECs on pilot/HSR protocols, in the absence of any other legal mechanism for assessment, researchers may be unable to satisfy a journal that a study protocol has received due independent consideration. One concern may be that researchers are being incentivized to treat this as an obstacle to overcome. There is some evidence that this has been viewed and institutionally recognized as a publication obstacle and needs somehow to be managed. Here is one such example:

“*When publishing the results of a quality control study, which does not require REK [the REC] approval, a researcher (author) can ask REK* *for a general statement for manuscript submission (“exempt from IRB (Institutional Review Board) evaluation”). This can be a convenient solution when you want to publish in a journal that requires all results to be based on a prior ethical review by the IRB. This is a requirement that all the reputable scientific journals have adopted and is based on Article 35 of the Helsinki Declaration*” [31].

Of course, not all journals have such stringent publication policies. In a brief review of recent articles in some Norwegian journals we found a report of an interventional study with no reference to ethical issues or whether independent ethics review had been obtained.

### Suggestions for future directions of travel

The 2017 legislation brings some change that may be welcomed. It may help to raise researcher standards through the provision for institutional committees to manage researcher misconduct. Decisions about prior approval may be appealed to the National Research Ethics Committee for Medicine and Health Sciences. However, it is still not clear what types of study regional RECs will, or should, consider. Moreover, there is no clear mechanism for alternative consideration in the cases where RECs consider the submitted protocol to be outside of their remit (rather than rejected).

Without improvements in these areas we are concerned that the issues identified in this paper may be set to continue. With new law focusing principally on managing misconduct at an institutional level, we are also concerned an association could emerge between cases that are considered by regional RECs to be outside of their remit, and subsequent misconduct cases brought at an institutional level against individual researchers. Apart from protecting the public, independent REC scrutiny may prevent researchers from making questionable judgments when moving to explore what may appear to be the next logical hypothesis, without first giving due consideration to all ethical issues. In this sense, independent committees provide a valuable service to researchers as well as to the wider public as the ultimate intended beneficiaries. It is important that access points and pathways for ethics support are made clear for researchers in Norway.

It would of course be an insurmountable task for regional committees to consider all undergraduate protocols. Legislative changes that clearly permits, encourages and provides for the existence of independent LRECs to address studies regional RECs consider outside of their remits, may avoid straining REC resources at the same time as ensuring a clear and practical mechanism for independent assessment of research considered to be outside of the regional RECs’ scope.

## Conclusion

We have argued that in HSR and some undergraduate health research in Norway, the *a priori* intent is to provide new knowledge of health and disease. Legally, regional Norwegian RECs must consider such study protocols. The effect of regional RECs declining to consider these may breed confusion, difficulty in managing ethical issues, and could risk public harm. New legislation provides mechanisms for institutional management of researcher misconduct but does not address variable interpretations of the Health Research Act and the regional RECs’ remit. To protect public health, we suggest it should either be made clear that assessing protocols for pilot studies and other HSR should be within the regional REC remit; or changes in legislation are needed to permit the existence of local RECs that can provide independent scrutiny in-line with international standards.

## Footnotes

1
*For this paper, we define LRECs as committees convened by institutions, such as universities and hospitals, that have a composition and independence that is consistent with the Declaration, and that may or may not be independent of a larger national ethics infrastructure*
